# Cement Burns: The Dublin National Burns Unit Experience

**Published:** 2007-10-24

**Authors:** Munir Alam, M. Moynagh, C. Lawlor

**Affiliations:** St James's Hospital, Dublin 8, Ireland

## Abstract

**Objective:** Cement burns account for relatively few admissions to a burn unit; however, these burns deserve separate consideration because of special features of diagnosis and management. Cement burns, even though potentially disabling, have rarely been reported in literature. **Methods:** A retrospective review was performed of all patients admitted with cement burns injuries to the national burns unit at the St James's Hospital in Dublin, Ireland, over a 10-year period for the years 1996–2005. **Results:** A total of 46 patients with cement burns were admitted. The majority of patients were aged 16–74 years (mean age = 32 years). Eighty-seven percent of injuries occurred in an industrial and 13% in a domestic setting. The upper and lower extremities were involved in all the patients, and the mean total body surface area affected was 6.5%. The mean length of hospital stay was 21 days with a range of 1–40 days. Thirty-eight (82%) were surgically managed involving debridement and split-thickness skin graft (SSG) and four (9%) were conservatively managed. A further four did not have data available. **Conclusion:** Widespread inexperience in dealing with this group of cement burns patients and delays in referral to burns unit highlights the potential for greater levels of general awareness and knowledge in both prevention and treatment of these burns. As well, early debridement and split-thickness skin grafting at diagnosis constitutes the best means of reducing the high socioeconomic costs and allows for early return to work.

Contacts with cements occurring in the industry or home are primarily accidental, and the costs of treatment tend to be high due to absence from the workplace. The lack of information and education regarding risks related to cement handling has been identified as a potential risk factor for this type of injury. Of many published articles, only a few rather recent publications report more substantial series,^1^–^4^ and notably in dermatological reviews mention only sporadic cases,^7^–^10^ and commensurate with the growth of construction industry there has been an increase in the frequency and magnitude of cement burns.

This is a review of our experience in the management of this patient population and highlights the need for improvements in education and early diagnosis and treatment of these costly injuries.

## MATERIAL AND METHODS

A 10-year retrospective study was conducted in the national burns unit at the St James's Hospital (SJH), Dublin, Ireland. All patients treated for cement burns between 1996 and 2005 were identified by review of meticulous record of inpatients kept by nursing staff within the unit.

Full medical records for these patients were then examined for age, sex, and occupation of the patients. Length of hospital stay, circumstances of the accident, type of cement used, length of exposure, site of injury, knowledge of risks, total body surface area (TBSA) affected, initial treatments, delay before medical and surgical management, follow-up, time to return to work, and possible occurrence of sequelae or residual problems were also examined. The distinction between domestic and industrial settings was made, and modes of treatment were either surgical or conservative.

## RESULTS

During the years 1996–2005, there were 2118 patients' admissions to the burns unit. Of these, 46 were admitted for cement burns, accounting for 2% of the total. Of those admitted, 45 were male and only 1 was female with a mean age of 32 years (range: 16–74 years) (Table 1).

No information regarding patients' occupations was given in 19 patients (41%), 20 patients (43%) had occupation within the construction industry or related area (construction workers, worker in cement plants, bricklayers, etc), and 7 patients (15%) belonged to other occupational groups.

Six (13%) of the cement burns cases reported occurred during do-it-yourself work, and 33 (71%) cases were work accidents. No information was available in 7 (15%) cases. The majority of cement burns affected the lower leg and ankles due to spilling of wet cement over the top of boots or the penetration of cement through defective protective shoes (36 cases, 78%). In these cases, it is notable that most patients did not take off their boots to clean them, but continued to wear them until finishing work, even if symptoms had already begun. Another frequent mode of injury was kneeling without additional protection for the knees, when leveling the concrete or cement, 5 (11%) of the cases described. Mean exposure time to cement was 60 minutes (1 min–24 hours). Only 5 (11%) patients had initial treatment with irrigation of the wound and 41 patients (89%) were treated with dressings ± antibiotics initially and later referred to the burn unit.

Thirteen (28%) patients presented to general practitioner on the same day, 6 (13%) on the following day, 2 (4%) after 2 days, and 15 (32%) presented more than 2 days with maximum delay up to 5 weeks, while 10 patients (22%) did not have data available.

The time to return to work after a mean of 4 months with average hospital stay was 21 days (range: 1–40 days) (Table 2).

The distribution of localization of injuries is shown in Table 3. It should be noted that some patients had burns to a single site and others to more than 1 site. The mean TBSA involved was 6.5% with a range of 0.5%–13% (Table 4).

Thirty-eight (82%) were surgically managed involving debridement and split-thickness skin graft (SSG), and 4 (9%) were conservatively managed. A further 4 patients did not have data available.

Most of the patients received delayed surgical treatment, and that was due to delay in referral to burns unit from the time of injury. However, all but 4 of these 46 patients were surgically treated relatively promptly within 4.5 days of admission.

## DISCUSSION

Records of the effect of cement on the skin date back to the 1700s and were presumed to be due to a contact dermatitis.^4^ Rowe and Williams first documented the true cement burn in 1963, which has since been well recognized.^4^

Cement burns have an insidious onset. Most patients comment that they notice only mild irritation initially. Cement contains lime (calcium oxide), which will potentially penetrate clothing and react with sweat causing an exothermic reaction. Even when not exposed to moisture, the dry powder is very hygroscopic and may also cause a desiccation injury. Hydrated calcium oxide becomes calcium hydroxide that causes skin damage primarily due to hydroxyl ion.^2^ If cement is not removed from the skin, it continues to corrode and often painlessly deepens necrosis under clothing. In emergency conditions, treatment of a cement burn should attempt to eliminate a maximum of toxic product by abundant washing of the wounds after removal of soaked clothing. Some authors recommend the application of a buffered phosphate solution to limit the spread of the product, but the wisdom of this practice is debatable, as the heat produced by the exothermic chemical reaction of neutralization could worsen the burns.^4^

The treatment of cement burns, though not unequivocal in the literature, is currently oriented toward early excision and grafting, once the diagnosis of full-thickness burns has been made.^3^,^4^,^6^

**Figure 1:**
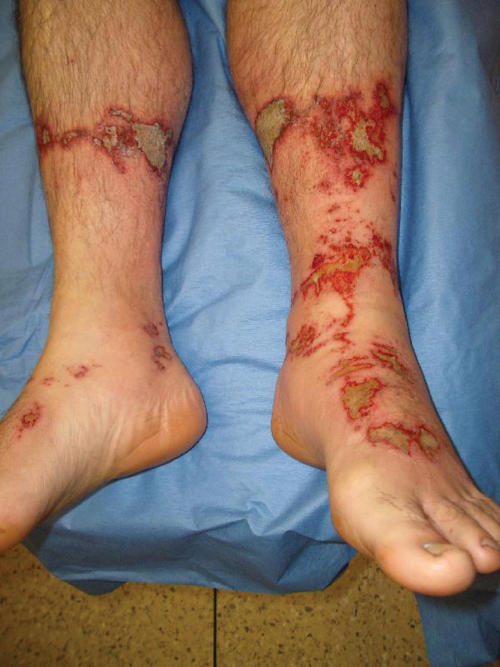
Appearance of cement burns on lower legs.

The anatomical distribution of cement burns to the lower leg, foot, and ankles (Fig 1) and to the knees is consistent with other studies.^4^ Floorers had burns on their knees from having to kneel while spreading cement and the added effect of pressure increasing the severity of the burns.^5^ Wearing short boots and Wellingtons were associated with ankle and calf burns, respectively.

These burns most commonly affect the extremities with localization especially in the lower limbs, notably on ankles, foot, and knees.^3^,^4^,^6^ This commonly involves a limited total BSA (rarely > 5%). Both of these factors were seen in our patient population.

Longer hospitalization of patients with cement burns was required for complete skin healing than in the overall burns group. This is consistent with previous reports.^2^ Unusually lengthy hospital stays for burns of relatively low TBSA were noted in the present series. In this group of patients, slow healing, graft failure, and regrafting are more common as compared to full-thickness lower extremity burns of different etiologies of the same area. As well, the depth of the burn requiring skin grafting and immobilization can also account for lengthy admissions to hospital.

Postburn sequelae include scar hypertrophy, skin fragility, and pruritis. These occur more frequently when healing is delayed for more than 3 weeks, as full-thickness burns, which are common in cement burns, do not heal by secondary intention. These sequelae can be avoided by early recognition of graft failure or inadequate debridement and any evidence of infection. After the healing period, postoperative follow-up should correspond to that of any burn graft, that is, prevention of the hypertrophic and contractile tendency of scars by the wearing of compression garments and massaging of the scars, possibly in association with physical therapy.^1^

## CONCLUSION

The incidence of cement burns has increased in our institution and now accounts for 5% of admissions to our burn unit over a 1-year period. This is due to recent increase in construction industry in Ireland and influx of workers from non–English-speaking European member states with language barrier making them vulnerable to be involved in cement-related accidents at construction sites.

The risks related to cement handling often go unrecognized. These burns can be avoided with good preventative education and early therapeutic management. In the case of any deep burns, surgical treatment at diagnosis constitutes the best means of reducing the high socioeconomic costs and early return to work.

## Figures and Tables

**Table 1:** Age distribution of patients with cement burns

**Age group, y**	**No. of patients**
10–19	2
20–29	16
30–39	13
40–49	4
50–59	8
60–75	2

**Table 2:** Length of hospital stay

	**Days**	**No. of patients**
1–9	8
10–19	22
20–29	8
30–40	7

**Table 3:** Anatomical site of cement burns

	**Anatomical site**	**No. of patients**
Forearms	3
Hands/wrists	7
Lower legs/knee	21
Feet/ankles	15

**Table 4:** Total body surface areas affected in patients*

	**TBSA, %**	**No. of patients**
<2	20
2–4	18
>4	3
Unknown	5

*TBSA indicates total body surface area.
